# Altered Expression of IFN-*λ*2 in Allergic Airway Disorders and Identification of Its Cell Origins

**DOI:** 10.1155/2016/5759496

**Published:** 2016-01-11

**Authors:** Qiuli Wang, Dong Chen, Hua Xie, Xiaoping Lin, Xuefeng Wang, Qijian Yao, Xiaoxuan Zheng, Chiyan Xu, Lingfei Chen, Shaoheng He, Huiyun Zhang

**Affiliations:** ^1^Department of ENT, Allergy and Clinical Immunology Research Centre, The First Affiliated Hospital of Liaoning Medical University, Jinzhou, Liaoning 121001, China; ^2^Department of Respiratory Medicine, The General Hospital of Shenyang Military Region, Shenyang, Liaoning 110016, China; ^3^Allergy and Inflammation Research Institute, Shantou University Medical College, Shantou 515031, China

## Abstract

This study investigated the expression levels of interferon- (IFN-) *λ*2 in peripheral blood and tissues. The results showed that the levels of IFN-*λ*2 were elevated by 17.9% and 14.2% in the plasma of allergic rhinitis (AR) and combined rhinitis with asthma (AR + AS), which was positively correlated with the level of tryptase but negatively correlated with the level of IL-10. IFN-*λ*2 was predominately expressed in the CD16+ cells and CD14+ cells in healthy control subjects (HC) but upregulated only in CD8+ cells of AR and in eosinophils of asthma. It was observed that approximately 6.6% and 7.0% dispersed tonsil cells and 5.8% and 0.44% dispersed lung cells are IFN-*λ*2+ mast cells and macrophages. Moreover, tryptase and agonist peptides of PAR-2 induced enhanced IFN-*λ*2 mRNA expression in A549 cells. In conclusion, the elevated levels of IFN-*λ*2 in the plasma of AR and AR + AS indicate that IFN-*λ*2 is likely to contribute to the pathogenesis of allergic airway disorders. The potential origins of the elevated plasma IFN-*λ*2 include mast cells, macrophages, and epithelial cells in tissues, neutrophils, monocytes, CD8+ T cells, and eosinophils in peripheral blood. Development of IFN-*λ*2 related therapy may help to treat or prevent allergic airway disorders.

## 1. Introduction

Human IFN-*λ*2 (IL-28A) is a relatively new cytokine, in which the genomic structure resembles that of the IL-10 family, but the protein structure is more closely related to type I IFN than to interleukin- (IL-) 10 [1, 2]. IFN-*λ*2 has been discovered to play a role in innate immunity. For example, it can induce antiviral activity in cell lines, though the potency is weaker than other IFNs [3], and has the potential antitumor effect against human lung cancer cells [4].

It has also been discovered that IFN-*λ*2 is capable of exacerbating T-cell-mediated autoimmune diseases such as uveitis [5]. Treatment with IFN-*λ*2 completely halts and reverses the development of collagen-induced arthritis, dramatically reduces the numbers of proinflammatory IL-17-producing Th17 and *γδ* T cells in the joints and inguinal lymph nodes, and restricts recruitment of IL-1b-expressing neutrophils [6]. However, IFN-*λ*2 seems not effective in inducing Tr1 cells [7] and cannot induce proliferation of regulatory T cells from cord blood CD4(+) T cells [8].

Recently, it was found that the expression level of IFN-*λ*2 mRNA was significantly increased during naturally occurring respiratory viral infections in children with asthma [9] and that IFN-*λ*2 modulates lung dendritic cells (DC) function to promote Th1 immune skewing and suppresses allergic airway disease [10]. These suggest that IFN-*λ*2 is not only involved in autoimmune diseases but also associated with allergic airway disorders. We therefore investigated the potential involvement of IFN-*λ*2 in allergic airway diseases in the present study.

To our surprise, information on the IFN-*λ*2 expressing cells is very limited. It was found that IFN-*λ*2 expressed in tracheobronchial tissue cells from the patients with COPD [11]. DC express moderate quantity of IFN-*λ*2 when using lipopolysaccharide (LPS) as the maturation stimulus [12], and vitiligo patient skin and/or peripheral blood mononuclear cells express IFN-*λ*2 mRNA [13]. In order to understand the role of IFN-*λ*2, we examined the cell origins of IFN-*λ*2 in the present study.

The aim of the study is to investigate the expression of IFN-*λ*2 in peripheral blood of allergic airway disorders, its correlation with cytokines and tryptase, and its potential cell location. We found that the levels of IFN-*λ*2 were elevated in the plasma of AR and AR + AS and that several cell types express IFN-*λ*2.

## 2. Materials and Methods

### 2.1. Reagents

Trypsin, leupeptin, collagenase (type I), hyaluronidase (type I), rabbit anti-human IFN-*λ*2 antibody, and bovine serum albumin (BSA, fraction V) were purchased from Sigma Aldrich (St. Louis, MO, USA). The sequences of the active and reverse peptides were PAR-2, trans-cinnamoyl-Leu-Ile-Gly-Arg-Leu-Orn-amide (tc-LIGRLO-NH_2_) and trans-cinnamoyl-Orn-Leu-Arg-Gly-Ile-Leu-amide (tc-OLRGIL-NH_2_), SLIGKV-NH_2_, and VKGILS-NH_2_; PAR-2 antagonist peptide Phe-Ser-Leu-Leu-Arg-Asn-NH_2_ (FSLLRN-NH_2_) was synthesized in CL Bio-Scientific Inc. (Xi'an, China). Dulbecco's modified Eagle's medium (DMEM) and fetal calf serum (FCS) were obtained from HyClone (Logan, UT, USA). Human tryptase ELISA kits were purchased from Cloud-Clone Corp. (Houston, USA). Human IFN-*λ*2, IL-4, IL-10, and IL-12 ELISA kits were purchased from R&D Systems (Minneapolis, MN). FOXP3 Fix/Perm Buffer Set, RBC Lysis Buffer (10x), FITC-anti-human CD123, PerCP-anti-human CD16, PerCP-anti-human HLA-DR, PerCP/Cy5.5-anti-human CD25, PerCP/Cy5.5-anti-human IL-17A, PE/Cy7-anti-human CD8, PE/Cy7-anti-human CD14, and PE/Cy7 conjugated rat anti-human IL-4 antibodies were purchased from BioLegend (San Diego, CA, USA). Lymphoprep was obtained from Axis-Shield Diagnostics Ltd. (Luna Place, Dundee, Scotland). Fixation/Permeabilization Solution Kit, FITC-anti-human CD4, APC-anti-human CD19, APC-anti-human IFN-*γ*, Alexa Fluor 647-anti-human FOXP3, and PE conjugated rat anti-mouse IgM antibodies were purchased from BD Pharmingen (San Jose, CA, USA). FITC or PE conjugated goat anti-rabbit IgG antibody was purchased from Santa Cruz Biotec (Santa Cruz, CA, USA). Rabbit IgG whole molecule was purchased from Rockland Immunochemicals Inc. (Limerick, Eire). Biotinylated rabbit anti-human IFN-*λ*2 was purchased from Bioss (Beijing, China). DAB + substrate chromogen system and ExtrAvidin-peroxidase conjugate were purchased from Chemicon International Inc. (Temecula, CA, USA). Anti-human tryptase antibody AA5 donated by Dr. A. F. Walls, University of Southampton, was conjugated with PE/Cy7. Anti-human chymase antibody CC4 (IgM subtype) was donated by Dr. A. F. Walls. Recombinant human lung *β*-tryptase was obtained from Promega (Madison, WI, USA). TRIzol Reagent was obtained from Invitrogen (Carlsbad, CA, USA). ExScript RT reagent kit and SYBR* Premix Ex Taq* (perfect real time) were obtained from TaKaRa Biotechnology Co., Ltd. (Dalian, China). Oligonucleotide primers for real-time PCR were synthesized by Invitrogen Biotechnology Co. (Shanghai, China). Allergens for skin prick were supplied by ALK-Abelló, Inc. (Denmark). Most of the general chemicals such as salts and buffer components were of analytical grade.

### 2.2. Patients and Samples

A total of 33 allergic rhinitis (AR), 26 asthma, 12 combined rhinitis with asthma (AR + AS), and 20 healthy control subjects (HC) were recruited in the study. The diagnosing criterion of asthma was conformed to the Global Initiative for Asthma [14], and diagnosis for allergic rhinitis was based on Allergic Rhinitis and its Impact on Asthma (ARIA) [15]. All patients were asked to stop antiallergy medication for at least 2 weeks prior to attending the study (those who could not stop antiallergy drugs were excluded). The recruited patients did not have any airway infection more than one month. The informed consent from each volunteer according to the Declaration of Helsinki and agreement with the Ethical Committee of the First Affiliated Hospital of Liaoning Medical University and General Hospital of Shenyang Military Area Command were obtained. The general characteristics of the patients and control subjects were summarized in Table 1. Peripheral venous blood sample (10 mL) was collected from each patient or HC and was immediately processed to collect cells and plasma for analysis. Specimens of human tissues for immunohistochemistry and flow cytometry analysis were collected from the Department of Pathology, The First Affiliated Hospital of Liaoning Medical University. Macroscopically normal lung tissue was removed at lobectomy from patients with carcinoma. Tonsillar tissue was removed at tonsillectomy. Nasal polyps were collected from AR patients. The protocol for ethical use of human tissue in research was according to the Declaration of Helsinki (2000) and approved by the Committees of the First Affiliated Hospital of Liaoning Medical University.

### 2.3. Flow Cytometry Examination of Expression of IFN-*λ*2 in Peripheral Blood Cells from Allergic Patients

To detect IFN-*λ*2 expression on leukocytes excluding T cells, the following antibodies were added to different testing tubes: (1) to detect IFN-*λ*2 expression in basophils: FITC-anti-human CD123 and PerCP-anti-human HLA-DR; (2) to detect IFN-*λ*2 expression in CD16+ polynucleated cells, CD16− polynucleated cells, and CD14+ cells and CD19+ cells: PerCP-anti-human CD16, PE/Cy7-anti-human CD14, and APC-anti-human CD19 before 200 *μ*L of whole blood being added at room temperature for 15 min in dark. Following ligation of red blood cells, white blood cells were fixed and permeabilized by using Cytofix/Cytoperm Fixation/Permeabilization Kit according to the manufacturer's instructions. Following washing with BD washing buffer, the cell pellets were resuspended and rabbit anti-human IFN-*λ*2 followed by PE or FITC conjugated goat anti-rabbit IgG antibodies were added at 4°C for 30 min. Finally, cells were resuspended in fluorescence-activated cell sorting- (FACS-) Flow solution and analyzed with FACSVerse flow cytometer (BD Biosciences, San Jose, CA). A total of 10,000 events were analyzed per population for each sample. Data were analyzed with CellQuest software (BD Immunocytometry systems).

For detection of IFN-*λ*2 expression in T cells, peripheral blood mononucleated cells (PBMC) were isolated by using Lymphoprep according to the manufacturer's instruction. The following antibodies were then added to different testing tubes: (1) FITC-anti-human CD4, PerCP/Cy5.5-anti-human CD25, PE/Cy7-anti-human CD8, and rabbit anti-human IFN-*λ*2 followed by Alexa Fluor 647-anti-human FOXP3 and PE conjugated goat anti-rabbit IgG antibodies to detect CD8+ T cells and regulatory T cells (Treg); (2) FITC-anti-human CD4, APC-anti-human IFN-*γ*, PE/Cy7 conjugated rat anti-human IL-4, PerCP/Cy5.5-anti-human IL-17A, and rabbit anti-human IFN-*λ*2 followed by PE conjugated goat anti-rabbit IgG antibodies to detect Th1, Th2, and Th17 cells. The FACS analysis procedure was similar to the one described above.

### 2.4. Dispersing Cells from Tissues and Flow Cytometry Analysis of IFN-*λ*2 Expression

The procedure for dispersing tissue cells was mainly adopted from the procedure described previously [16]. Cells were then incubated with each labeled monoclonal antibody including (1) PE/Cy7 conjugated mouse anti-human tryptase, anti-human chymase antibody CC4 (IgM subtype), PE conjugated rat anti-mouse IgM, rabbit anti-human IFN-*λ*2, and FITC conjugated goat anti-rabbit IgG antibodies to detect mast cells; (2) PE/Cy7-anti-human CD14, APC-anti-human CD19, rabbit anti-human IFN-*λ*2, and FITC conjugated goat anti-rabbit IgG antibodies to detect macrophages and B cells at 4°C for 30 min in dark. After washing, the cell pellets were resuspended in FACS-Flow solution and analyzed with FACSVerse flow cytometer. A total of 10,000 events were analyzed for each sample. Data were analyzed with CellQuest software.

### 2.5. Immunocytochemical Staining of IFN-*λ*2 in Human Tissues

Tissues were fixed in Carnoy's fixative, dehydrated, and embedded in paraffin wax. Sections (4 *μ*m) were dewaxed, rehydrated, and incubated for 10 min with 0.5% H_2_O_2_ in methanol followed by 0.1% sodium azide for 10 min in order to inhibit endogenous peroxidase activity. PBS containing 5% BSA was added for 1 h and the same solution was employed as the diluent for the antibodies added subsequently. Sequential sections of tonsil, lung, or nasal polyps were incubated with biotinylated rabbit anti-human IFN-*λ*2 for 2 h. After washing with PBST, ExtrAvidin-peroxidase conjugate was applied to sections for 1 h. Staining was developed over 4 min by using DAB chromogen system before being counterstained with Mayer's haematoxylin and mounted in AquaMount. For each section, the number of positively stained cells was counted in at least 30 fields (the area of each field equals 0.19 mm^2^). A single-blind method was used to examine the slides.

### 2.6. A549 Cell Culture and Challenge

The human lung carcinoma cell line A549 (morphology: epithelial) was obtained from the American Type Culture Collection (Manassas, VA, USA). Cells were grown in Dulbecco's modified Eagle's medium (DMEM), supplemented with 10% (v/v) fetal calf serum (FCS), 100 U/mL penicillin, and 100 *μ*g/mL streptomycin. Cells were cultured in 75 cm^2^ tissue culture flasks (Falcon) at 37°C in a 5% (v/v) CO_2_, water-saturated atmosphere.

For challenge experiments, cells were detached from culture flasks using trypsin, seeded into 12-well cell culture plates, and grown to about 80% confluence. The cells were then cultured with the serum-free basal medium for an additional 16 h before challenge. Cells were exposed to tryptase (2 *μ*g/mL, 1 *μ*g/mL = 7.4 nM) with or without its inhibitor leupeptin (3 *μ*g/mL), 100 *μ*M of SLIGKV-NH_2_ with or without PAR-2 antagonist FSLLRN-NH_2_ (400 *μ*M) and its reverse peptide VKGILS-NH_2_, and 100 *μ*M of tc-LIGRLO-NH_2_ with or without PAR-2 antagonist FSLLRN-NH_2_ (400 *μ*M) and its reverse peptide tc-OLRGIL-NH_2_, respectively. Cells (1.5 × 10^6^ per well) were collected at 2 h or 6 h, centrifuged at 4°C, and stored at −80°C until use.

### 2.7. Quantitative Real-Time PCR (qPCR) Analysis of IFN-*λ*2 mRNA Expression in A549 Cells

The expression of IFN-*λ*2 mRNA in A549 cells was determined by qPCR following the manufacture's protocol. Briefly, after synthesizing cDNA from total RNA by using Superscript first strand synthesis system for RT-PCR and oligo-dT primers, real-time PCR was performed by using SYBR* Premix Ex Taq* kit on the ABI Prism 7700 Sequence Detection System (Perkin Elmer Applied Systems, Foster City, CA, USA). Sequence-specific standard curves were generated using 10-fold serial dilutions of plasmid DNA, and the values for the initial concentrations of unknown samples were calculated by using the software (version 1.7) provided with the ABI 7700 system. IFN-*λ*2 mRNA expression in each sample was finally determined after correction with *β*-actin expression. Each measurement of a sample was conducted in duplicate. The primers for IFN-*λ*2 were forward: 5′-CACCCTGCACCATATCCTCT-3′, reverse: 5′-GGAGGGTCAGACACACAGGT-3′ and for *β*-actin were forward: 5′-AGAGCTACGAGCTGCCTGAC-3′, reverse: 5′-AGCACTGTGTTGGCGTACAG-3′.

### 2.8. Determination of Levels of Tryptase and Cytokines

Levels of tryptase, IL-4, IL-10, IL-12, and IFN-*λ*2 in the plasma of AR, asthma, AR + AS, and HC were measured by using ELISA kits according to the manufacturer's instructions.

### 2.9. Statistical Analysis

Statistical analyses were performed by using SPSS software (Version 13.0). Data were expressed as mean ± SEM. Where analysis of variance indicated significant differences between groups with ANOVA, Student's *t*-test was applied. Data for allergic patients are presented as scatter plot. Where Kruskal-Wallis analysis indicated significant differences between groups, for the preplanned comparisons of interest, the paired Mann-Whitney *U* test was employed. Correlations were determined using Spearman rank correlation. For all analyses, *P* < 0.05 was taken as significant.

## 3. Results

### 3.1. Levels of IFN-*λ*2 in the Patients with Allergic Rhinitis and Asthma and Its Correlation with Tryptase and Cytokines

**Figure 1:**
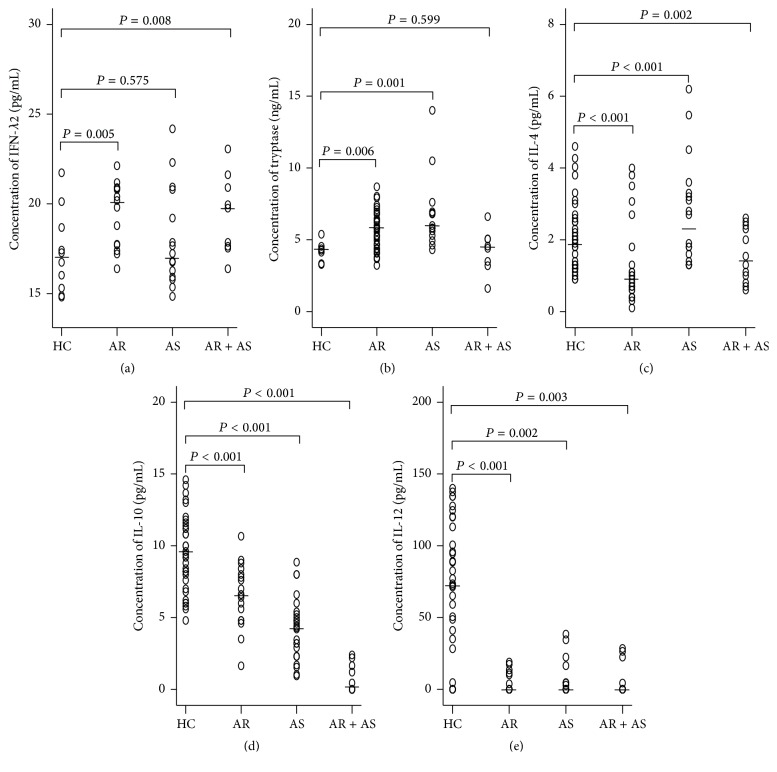
Scatter plots of the levels of IFN-*λ*2 (a), tryptase (b), IL-4 (c), IL-10 (d), and IL-12 (e) in the plasma of the patients with allergic rhinitis (AR), asthma (AS), and combined AR and asthma (AR + AS) and healthy control subjects (HC). Each symbol represents the value from one subject. The median value is indicated with a horizontal line. *P* < 0.05 was taken as statistically significant.

In order to evaluate the potential role of IFN-*λ*2 in allergic airway disorders, the most direct evidence is to examine the changes of its levels in clinical specimen. We therefore examined the levels of IFN-*λ*2 in the plasma and its cellular location in blood of the patients with AR and asthma. The results showed that the levels of IFN-*λ*2 were elevated by 17.9% and 14.2% in the plasma of AR and combined rhinitis with asthma (AR + AS), but not of asthma (Figure 1(a)). The plasma levels of tryptase were increased by 34.7% and 38.3% in the patients with AR and asthma, but not AR + AS (Figure 1(b)). The plasma levels of IL-4 were increased by 21.1% in the patients with asthma but decreased by 55.3% and 26.3% in AR and AR + AS (Figure 1(c)). The plasma levels of IL-10 (Figure 1(d)) and IL-12 (Figure 1(e)) were decreased by 29.8% and 100% in the patients with AR, by 54.3% and 100% in the patients with asthma, and by 100% and 100% in the patients with AR + AS, respectively. There were positive correlation between IFN-*λ*2 and tryptase and negative correlation between IFN-*λ*2 and IL-10 in the plasma of AR. Similarly, plasma IFN-*λ*2 positively correlates with tryptase, and IL-10 positively correlates with IL-12 in asthma (Table 2).

### 3.2. Expression of IFN-*λ*2 in Peripheral Blood Leukocytes

**Figure 2:**
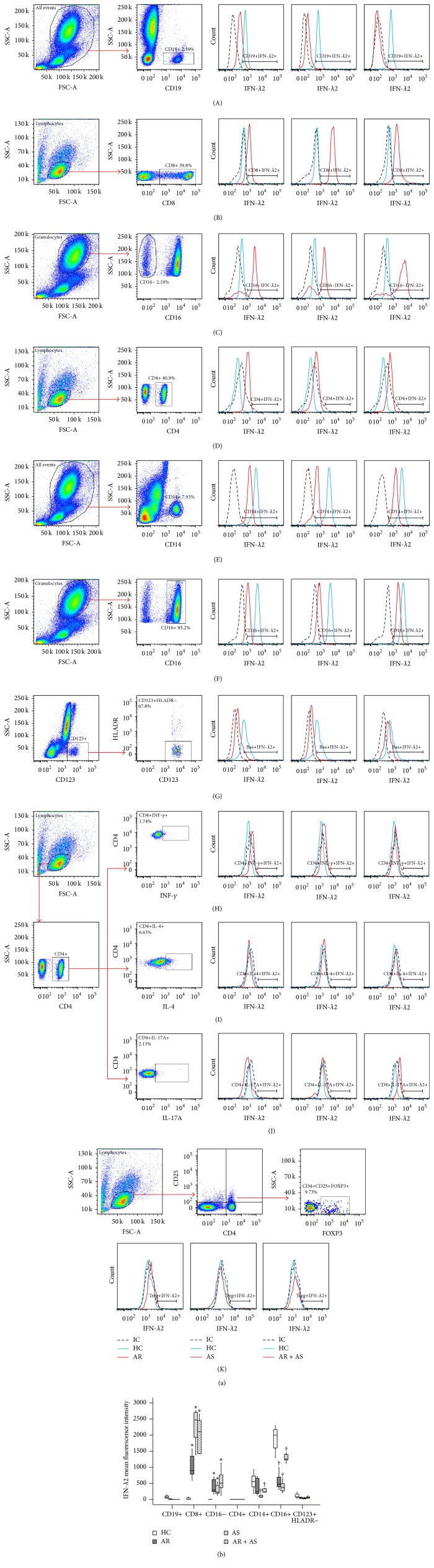
Flow cytometry analysis of the expression of IFN-*λ*2 in peripheral blood leukocytes. (a) (A), (B), (C), (D), (E), (F), (G), (H), (I), (J), and (K) showed a representative graph of the changes in mean fluorescent intensity in CD19+ cells (B cells), CD8+ cells (cytotoxic T cells), CD16− polynucleated cells (eosinophils), CD4+ cells (helper T cells), CD14+ cells (monocytes), CD16+ cells (neutrophils), CD123+HLA-DR− cells, CD4+INF-*γ*+ cells (Th1 cells), CD4+IL-4+ cells (Th2 cells), CD4+IL-17A+ cells (Th17 cells), and CD4+CD25+FOXP3+ cells (regulatory T cells) in allergic rhinitis (AR), asthma (AS), and combined AR and AS (AR + AS) and healthy control subjects (HC), respectively. IC = isotype control. (b) represents boxplot analysis of the expression of IFN-*λ*2 in peripheral blood leukocytes. Data are displayed as a boxplot for AR (*n* = 33), AS (*n* = 26), AR + AS (*n* = 12), and HC (*n* = 20), which indicates the median, the interquartile range, and the largest and smallest values for the number of subjects indicated. ^*∗*^ *P* < 0.05, increased value compared with HC. ^†^ *P* < 0.05, decreased value compared with HC.

In order to identify the potential sources of IFN-*λ*2, we investigated the expression of IFN-*λ*2 in peripheral blood leukocytes. The results showed that IFN-*λ*2 was predominately expressed in the CD16+ (representing neutrophils) (Figure 2(a)(F)) and CD14+ cells (representing monocytes) (Figure 2(a)(E)) and weakly expressed in CD19+ (representing B cells) (Figure 2(a)(A)), CD8+ cells (representing cytotoxic T cells) (Figure 2(a)(B)), and basophils (Figure 2(a)(G)). CD4+ T cells (Figure 2(a)(D)) and CD16− polynucleated cells (representing eosinophils) (Figure 2(a)(C)) seemed not to express IFN-*λ*2 in HC (Figure 2(b)). However IFN-*λ*2 expression was upregulated by 43.5% and 49.1% in AR, by 125% and 42.3% in asthma, and by 99% and 72.8% in AR + AS in cytotoxic T cells and eosinophils but downregulated by 57% and 76.3% in AR, by 86.4% and 81.6% in asthma, and by 58.1% and 37.2% in AR + AS in monocytes and neutrophils, respectively (Figure 2(b)).

### 3.3. Immunohistochemical Staining of IFN-*λ*2 in Tissue Cells

**Figure 3:**
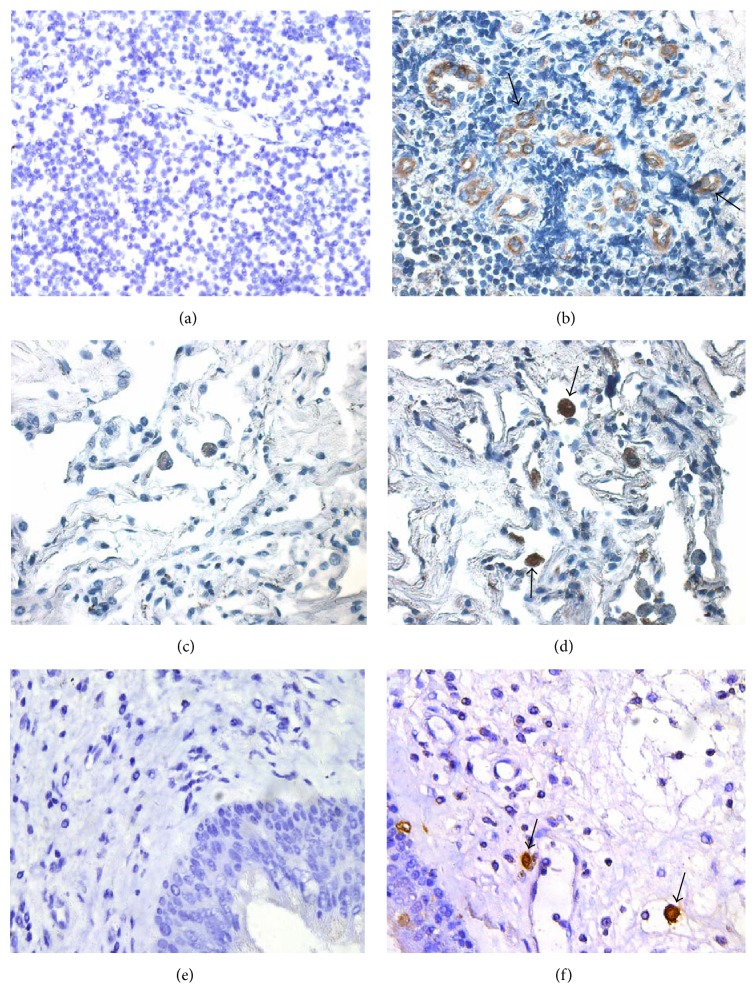
Immunohistochemical staining of IFN-*λ*2 in human tonsil, lung, and nasal polyps tissues. Biotinylated anti-human IFN-*λ*2 antibody was employed to identify IFN-*λ*2+ cells in the sections from tonsil (b), lung (d), and nasal polyps (f), respectively. Mouse IgG-peroxidase labeled anti-mouse IgG was used as negative control for tonsil (a), lung (c), and nasal polyps (e). Arrows indicate positively stained cells. All sections were counterstained with Mayer's haematoxylin. Magnification was ×400.

In order to further investigate the potential source of IFN-*λ*2, we examined the expression of IFN-*λ*2 in cells of various tissue origins by using immunohistochemical staining technique. The results showed that IFN-*λ*2 clearly expresses in glandular epithelial cells and some large cells (most likely mast cells or macrophages) in tonsillar tissue (Figure 3(b)) and in some large cells in lung tissue (Figure 3(d)) and nasal polyps (Figure 3(f)) as compared with the negative control tissues (Figures 3(a), 3(c), and 3(f)).

### 3.4. Expression of IFN-*λ*2 in Airway Tissues and Cells

**Figure 4:**
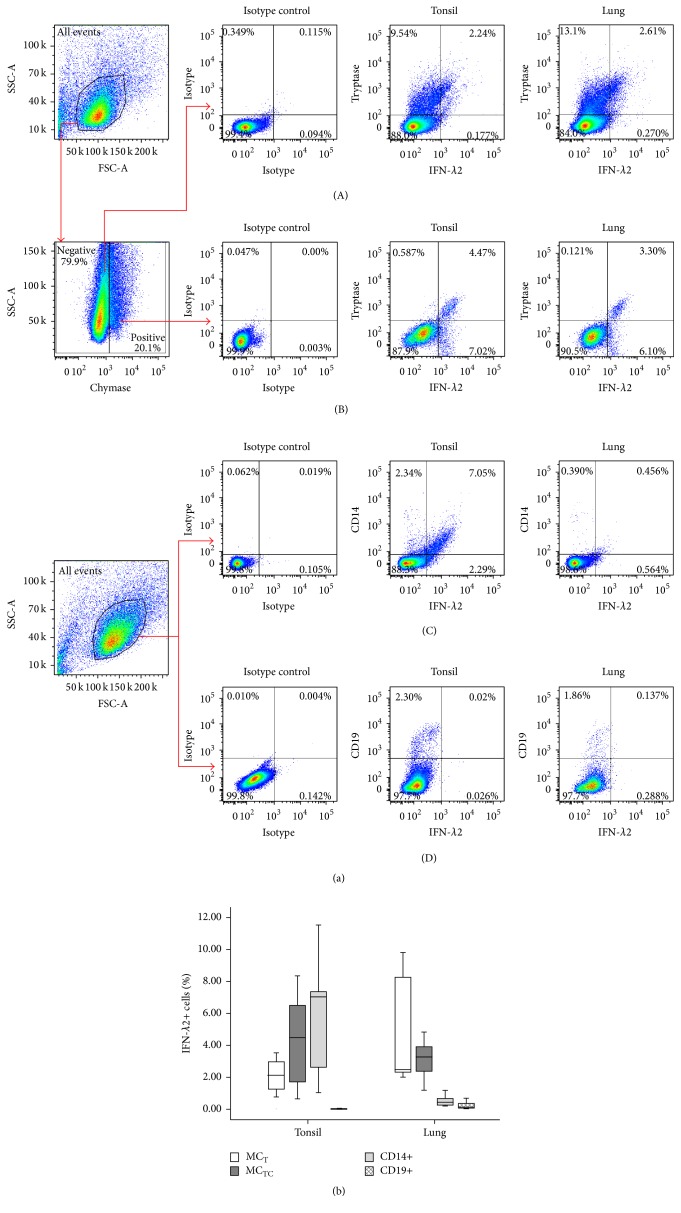
Flow cytometry analysis of expression of IFN-*λ*2 in dispersed human MC_T_, MC_TC_, CD14+ (macrophages), and CD19+ (B cells) cells. Rabbit anti-human IFN-*λ*2 and FITC conjugated goat anti-rabbit IgG antibodies were employed to detect IFN-*λ*2 in cells from tonsil and lung, respectively. PE/Cy7-anti-human tryptase (AA5-PE/Cy7), anti-human chymase antibody CC4 (IgM subtype), PE conjugated rat anti-mouse IgM (CC1-PE), PE/Cy7-anti-human CD14 (CD14-PE/Cy7), and APC-anti-human CD19 (CD19-APC) antibodies were used to identify MC_T_ (A), MC_TC_ (B), CD14+ (C), and CD19+ cells (D), respectively. (a) showed representative graphs of flow cytometry analysis. (b) represents boxplot analysis of the expression of IFN-*λ*2 in dispersed human MC_T_, MC_TC_, CD14+, and CD19+ cells. Data are displayed as a boxplot for 6-7 human tonsil or lung tissues, which indicates the median, the interquartile range, and the largest and smallest values for the number of subjects indicated.

To confirm the immunohistochemical staining observations, we examined IFN-*λ*2 expression in dispersed human tonsil and lung mast cells, B cells, and macrophages by flow cytometry analysis. The results showed that approximately 2.1%, 4.5%, and 7.0% dispersed tonsil cells are IFN-*λ*2+ MC_T_ mast cells, MC_TC_ mast cells, and macrophages. B cells did not express IFN-*λ*2. In lung tissue, however, 2.5%, 3.3%, 0.44%, and 0.14% dispersed cells are IFN-*λ*2+ MC_T_ mast cells, MC_TC_ mast cells, macrophages, and B cells (Figure 4).

### 3.5. Induction of the Expression of IFN-*λ*2 mRNA in A549 Cells

**Figure 5:**
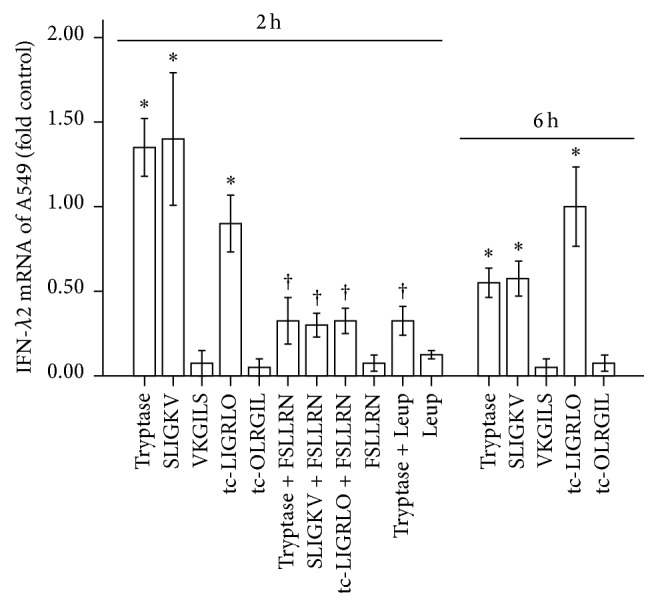
Induction of the expression of IFN-*λ*2 mRNA in A549 cells by tryptase and agonist peptides of PAR-2. Cells were treated with tryptase (2.0 *μ*g/mL) with or without leupeptin (Leup, 3.0 *μ*g/mL), SLIGKV-NH_2_ (SLIGKV, 100 *μ*M) with or without FSLLRN-NH_2_, reverse peptide VKGILS-NH_2_ (VKGILS, 100 *μ*M), tc-LIGRLO-NH_2_ (tc-LIGRLO, 100 *μ*M) with or without FSLLRN-NH_2_ (FSLLRN, 400 *μ*M), and reverse peptide tc-OLRGIL-NH_2_ (tc-OLRGIL, 100 *μ*M). Total cellular RNA was isolated and reversely transcribed to cDNA, and the cDNA was used for quantitative real-time PCR analysis. The data were normalized to the housekeeping gene (*β*-actin gene) and were expressed as mean ± SE fold of control for four separate experiments performed in duplicate. ^*∗*^ *P* < 0.05 compared with the baseline control. ^†^ *P* < 0.05 compared with the response to the corresponding stimulus alone.

Positive correlation of IFN-*λ*2 with tryptase implicated that the increased level of IFN-*λ*2 in the plasma of patients with AR and AR + AS may be elicited by mast cell tryptase. To confirm this anticipation, we examined the effect of tryptase and agonist peptides of PAR-2 on IFN-*λ*2 mRNA expression in A549 cells. It was found that the expression of IFN-*λ*2 mRNA over baseline control was increased by approximately 1.4- and 0.5-fold when the cells were incubated with tryptase at 2 *μ*g/mL for 2 and 6 h (Figure 5). Similarly, SLIGKV-NH_2_ and tc-LIGRLO-NH_2_ induced approximately 1.4- and 0.9-fold increase in expression of IFN-*λ*2 mRNA over baseline control, respectively, when they were incubated with A549 cells for 2 h (Figure 5). At 6 h following incubation with SLIGKV-NH_2_ and tc-LIGRLO-NH_2_, the expression of IFN-*λ*2 mRNA was enhanced by approximately 0.6- and 1.0-fold, respectively (Figure 5). The reverse peptides VKGILS-NH_2_ and tc-OLRGIL-NH_2_ showed little effect on the expression of IFN-*λ*2 mRNA in A549 cells following 2 and 6 h incubation periods (Figure 5).

Since FSLLRN-NH_2_ and leupeptin were able to inhibit tryptase induced upregulation of expression of IFN-*λ*2 mRNA and FSLLRN-NH_2_ suppressed SLIGKV-NH_2_ and tc-LIGRLO-NH_2_ induced upregulation of IFN-*λ*2 mRNA expression (Figure 5), the action of tryptase is likely to be mediated by PAR-2 and requires its enzymatic activity.

## 4. Discussion

We have demonstrated for the first time that the levels of IFN-*λ*2 are elevated in plasma of the patients with AR and AR + AS, but not with asthma, which provides the first hard evidence for proving that IFN-*λ*2 may participate in adoptive immune response such as allergic airway reactions. The recent reports that the expression level of IFN-*λ*2 mRNA was significantly increased during naturally occurring respiratory viral infections in children with asthma [9] and that IFN-*λ*2 was capable of exacerbating a T-cell-mediated autoimmune disease [5] may support our observation.

It is difficult to evaluate the role of IFN-*λ*2 in allergic airway inflammation at this stage as we do not know if the increased serum level of IFN-*λ*2 is a causative or resulting factor in the pathogenesis of the allergic airway disorders. Our observation that elevated IFN-*λ*2 levels were positively correlated to tryptase level in the plasma of AR suggests that these two compounds are likely released from the same source. Since tryptase is a relatively selective marker of mast cell degranulation and the most abundant secretory product from mast cells [17], it is likely that IFN-*λ*2 is also released from mast cells upon degranulation. Indeed, we have demonstrated in the present study that large numbers of tonsil and lung MC_T_ and MC_TC_ subtypes of mast cells express IFN-*λ*2, confirming that mast cells are the major source of IFN-*λ*2. Our previous report that IFN-*λ*1 (IL-29) highly expressed in mast cells [18] may support our current observation.

However, unlike tryptase acting as a potent proinflammatory mediator which is capable of provoking microvascular leakage in the skin of guinea pigs [19], stimulating the release of histamine from dispersed human tonsil mast cells [16], and inducing accumulation of eosinophils and neutrophil in the peritoneum of mice [20], IFN-*λ*2 appears to act as a suppressor of allergic airway diseases. For example, IFN-*λ*2 can modulate lung DC function to promote Th1 immune skewing and suppress allergic airway disease [10]. Since the information on the role of IFN-*λ*2 in allergy is very limited, the study that treatment with IFN-*λ*2 completely halts and reverses the development of collagen-induced arthritis, dramatically reduces numbers of proinflammatory IL-17-producing Th17 and *γδ* T cells in the joints and inguinal lymph nodes, and restricts recruitment of IL-1b-expressing neutrophils [6] may support the anticipation that IFN-*λ*2 may play an inhibitory role in allergic airway diseases.

Since a large population of macrophages express IFN-*λ*2, it is likely one of major sources of IFN-*λ*2, considering huge numbers of macrophages in lung and tonsil. Epithelial cells could be another source of IFN-*λ*2 as tonsil glandular epithelial cells express IFN-*λ*2, and A549 cells express IFN-*λ*2 mRNA. Our observation that tryptase induced upregulation of expression of IFN-*λ*2 mRNA in A549 cells is mediated by PAR-2 and requires tryptase enzymatic activity implicates that tryptase may provoke IFN-*λ*2 production in lung epithelial cells through activation of PAR-2, and released IFN-*λ*2 could contribute to the elevated plasma level of IFN-*λ*2 in allergic airway disorders. Obviously, further work is required to prove this speculation. Since little is known of the relationship between PARs and IFN-*λ*s, our previous report that the actions of thrombin on A549 cells are most likely carried out through hydrolytic cleavage of N-terminal of PAR-1 [21] may help to understand our observation above.

We have also observed the declined plasma levels of IL-10 and IL-12 in the allergic patients. Since the correlation between IL-12 and IL-10 levels in serum has been reported in the patients with atopic dermatitis [22], and diminished IL-12 levels were previously found in the serum of allergic patients [23], our observation may further suggest that reduced IL-10 and IL-12 production may contribute to the pathogenesis of the airway allergic disorders. The negative correlation between IFN-*λ*2 and IL-10 in the plasma of AR suggested they are not likely to be released from same sources, which means that if mast cells are major source of IFN-*λ*2, they should not be the major source for IL-10 in AR.

In order to identify the potential source of increased IFN-*λ*2, we investigated the expression of IFN-*λ*2 in peripheral blood leukocytes. Our data showed that IFN-*λ*2 expression was downregulated in AR, in asthma, and in AR + AS in monocytes and neutrophils. Since neutrophils and monocytes are predominant IFN-*λ*2-expressing cells in blood of HC, the decreased expression of IFN-*λ*2 in these 2 cell types could contribute to diminished level of IFN-*λ*2 in the plasma of asthma, even though IFN-*λ*2 expression appeared to be upregulated in blood cytotoxic T cells and eosinophils in asthma as cytotoxic T cells only weakly express and eosinophils do not express IFN-*λ*2 in HC. Downregulation of expression of IFN-*λ*2 in peripheral blood monocytes and neutrophils of AR and AR + AS seemed to conflict with the observation of increased level of IFN-*λ*2 in the plasma of AR and AR + AS, which suggests that there must be some other sources to generate large amount of IFN-*λ*2 apart from blood leukocytes. Moreover since helper T cells including regulatory T cells do not express IFN-*λ*2, they are one of the major sources of IL-10, which may at least partially explain the negative correlation between IFN-*λ*2 and IL-10 in the plasma of AR.

## 5. Conclusion

In conclusion, the elevated levels of IFN-*λ*2 in the plasma of AR and AR + AS and positive correlations of plasma IFN-*λ*2 with tryptase in AR and asthma indicate that IFN-*λ*2 is likely to contribute to the pathogenesis of allergic airway disorders. Mast cells, macrophages, and epithelial cells in human tonsil and lung tissues express IFN-*λ*2, and upregulated IFN-*λ*2 expression was observed in CD8+ T cells and eosinophils of allergic airway disorders indicate that they are the potential sources of IFN-*λ*2. Development of IFN-*λ*2 related therapy may help to treat or prevent allergic airway disorders.

## Figures and Tables

**Table 1:** General characteristics of the patients with rhinitis (AR), asthma (AS), and combined rhinitis with asthma (AR + AS) or healthy control subjects (HC).

	HC	AR	AS	AR + AS
	(*n* = 20)	(*n* = 33)	(*n* = 26)	(*n* = 12)
Age (yr)	38 (17–69)	41 (17–78)	46 (17–75)	43 (17–59)
Female/male	12/8	19/14	13/13	7/5
Median age at onset (yr)	na	37 (7–58)	41 (10–57)	45 (6–58)
Median disease duration (yr)	na	3 (0.5–20)	3.5 (0.5–40)	5 (1–38)
Blood taken time after the first symptom of the latest attack (h)	na	13 (4–20)	9 (3–16)	10 (3–20)
Number of positive skin pricks for dust mite	0	17	15	7
Number of positive skin pricks for artemisia	0	6	4	3
Number of positive skin pricks for ragweed	0	2	7	0
Number of positive skin pricks for cat fur	0	5	3	2
Number of positive skin pricks for dog fur	0	5	2	1

Median (range) data are shown for the number of subjects indicated. All patients stop using long-acting corticosteroids for at least two weeks and any other antiallergic drugs for one week before skin prick being taken. na = not applicable.

**Table:** (a) In the patients with allergic rhinitis

	IFN-*λ*2	Tryptase	IL-4	IL-10	IL-12
IFN-*λ*2	1	0.613187^*∗*^	−0.168899	−0.606208^*∗*^	—
Tryptase	0.613187^*∗*^	1	−0.435526^*∗*^	−0.304311	0.153744
IL-4	−0.168899	−0.435526^*∗*^	1	0.360302	0.370014
IL-10	−0.606208^*∗*^	−0.304311	0.360302	1	0.388322
IL-12	—	0.153744	0.370014	0.388322	1

^*∗*^Correlation is significant at the 0.05 level.

**Table:** (b) In the patients with asthma

	IFN-*λ*2	Tryptase	IL-4	IL-10	IL-12
IFN-*λ*2	1	0.463068^*∗*^	0.030461	0.316169	—
Tryptase	0.463068^*∗*^	1	0.34891	0.194955	—
IL-4	0.030461	0.34891	1	0.169851	—
IL-10	0.316169	0.194955	0.169851	1	0.767213^*∗*^
IL-12	—	—	—	0.767213^*∗*^	1

^*∗*^Correlation is significant at the 0.05 level.

**Table:** (c) In the patients with combined allergic rhinitis with asthma

	IFN-*λ*2	Tryptase	IL-4	IL-10	IL-12
IFN-*λ*2	1	0.214286	−0.066667	0.118818	—
Tryptase	0.214286	1	0.47619^*∗*^	−0.109109	—
IL-4	−0.066667	0.47619^*∗*^	1	0.322193	−0.305699
IL-10	0.118818	−0.109109	0.322193	1	—
IL-12	—	—	−0.305699	—	1

^*∗*^Correlation is significant at the 0.05 level.
